# Impact Factor Insights 2025: Analysis of Pakistani Academic Journals

**DOI:** 10.12669/pjms.41.7.12693

**Published:** 2025-07

**Authors:** Sultan Ayoub Meo, Shaukat Ali Jawaid

**Affiliations:** 1 Prof. Sultan Ayub Meo, MBBS, Ph. D, FRCP (London, Dublin, Glasgow, Edinburgh) Professor and Consultant, Department of Physiology and Clinical Physiology, College of Medicine, and King Khalid University Hospital, King Saud University, Riyadh, Saudi Arabia; 2 Shaukat Ali Jawaid Chief Editor, Pakistan Journal of Medical Sciences, Karachi - Pakistan

On June 18, 2025, the highly reputable global organization, Clarivate, released the Journal Citation Report (JCR) 2025, an international ranking list of scholarly journals. This report provides comprehensive information about the journals indexed in the Science Citation Index Expanded (SCIE), the Web of Science Core Collection, and the Emerging Sources Citation Index (ESCI). Clarivate has indexed 22,249 journals from 111 countries across 254 categories. This includes 14,591 science journals, 7,559 social science journals, 3,368 arts and humanities journals, and 618 journals that received their first Journal Impact Factor.^1^

Similar to year 2024, CA-A CANCER JOURNAL FOR CLINICIANS (232.4), Nature Reviews Drug Discovery (101.8), Lancet (88.5), New England Journal of Medicine (78.5), British Medical Journal (42.7). Nature (48.5) and Science (45.8) ranked high in the worldwide Impact Factor race. These are highly reputable journals worldwide, and publishing articles in them is the dream of academicians, researchers and policymakers. However, among these top journals, some journals impact factor is decreased such as in year 2024 the Impact Factor of CA-A CANCER JOURNAL FOR CLINICIANS was (521.6), Nature Reviews Drug Discovery (122.8), Lancet (98.4), New England Journal of Medicine (96.3), British Medical Journal (93.7). Nature (50.5)^1^,^2^. The journal’s impact factor declined in the recent 2025 report. These journals are highly reputable and top-ranked and are involved in global guidelines and policies.

According to the academic journals from Pakistan listed by Clarivate 2025, only 33 journals secured a position in the Web of Science. However, 11 of these journals are indexed in the Science Citation Index Expanded (SCIE) Core Collection, and 19 journals appeared in the Emerging Sources Citation Index (ESCI) (Table-I). These 19 journals in the ESCI are still under investigation, and it remains unclear whether they will be included in the core collection or dropped (Table-I). In the Clarivate, SCIE, Report 2025, two journals were ranked in quartile rankings One and Two. Pakistan Veterinary Journal, with an IF of 5.4 and a quartile ranking of One, and Pakistan Journal of Medical Sciences, with an IF of 1.7 and a quartile ranking of Two. The remaining 09 journals had an IF of less than 1.0.

**Table-I:** Academic Journals from Pakistan indexed in Clarivate with Impact Factor and Quartile Ranking

Name of the Journal	Impact Factor 2024	Quartile Ranking 2024	Impact Factor 2025	Quartile Ranking 2025
***Journals Indexed in Science Citation Index Expanded (SCIE)***				
1.Pakistan Veterinary Journal	3.8	Q1	5.4	Q1
**2. Pakistan Journal of Medical Sciences**	**1.2**	**Q2**	**1.7**	**Q2**
3. Journal of the Pakistan Medical Association	0.8	Q3	0.8	Q3
4. Journal of the College of Physicians and Surgeons Pakistan	0.7	Q3	0.8	Q3
5. Pakistan Journal of Agricultural Sciences	0.7	Q3	0.6	Q3
6. Pakistan Journal of Botany	0.9	Q4	0.9	Q4
7. Journal of the Chemical Society of Pakistan	0.6	Q4	0.5	Q4
8. Pakistan Journal of Pharmaceutical Sciences	0.7	Q4	0.6	Q4
9. Pakistan Journal of Zoology	0.5	Q4	0.5	Q4
10. Journal of Animal and Pant Science- JAPS	0.6	Q3	0.5	Q4
11. International Journal of Pharmacology	0.3	Q4	0.2	Q4
***Journals indexed in Emerging Sources Citation Index (ESCI)*** ***Note:** These journals are still under evaluation and have not shifted to SCIE*				
1. Pakistan Journal of Statistics & Operations Research	1.1	Q3	0.8	Q3
2. Annals of King Edward Medical University, Lahore	0.1	Q4	0.1	Q4
3. Pakistan Heart Journal	0.2	Q4	0.4	Q4
4. Pakistan Journal of Analytical & Environmental Chemistry	0.4	Q4	0.4	Q4
5. Journal of the Pakistan Institute of Chemical Engineers	0.1	Q4	0.1	Q4
6. Rawal Medical Journal	0.4	Q4	0.2	Q4
7. Annals Abbasi Shaheed Hospital & Karachi Medical & Dental College	0.1	Q4	0.1	Q4
8. Gomal Journal of Medical Sciences	0.5	Q4	0.1	Q4
9. Journal of the Liaquat University of Medical and Health Sciences	0.2	Q4	0.2	Q4
10. Khyber Medical University Journal-KMUJ	0.2	Q4	0.2	Q4
11. FWU Journal of Social Sciences	0.8	Q3	1	Q3
12. Discrete Mathematics Letters	1.0	Q3	0.8	Q3
13. Advancements in Life Sciences	0.9	Q3	0.8	Q3
14. Mehran University Research Journal of Engineering and Technology	0.6	Q3	0.7	Q3
15 Anesthesia Pain and Intensive Care	0.2	Q4	0.3	Q4
16. Soil and Environment	0.4	Q4	0.3	Q4
17. IPRI Journal-Islamabad Policy Research Institute	0.2	Q4	0.2	Q4
18. Asian Journal of Management Cases	0.1	Q4	0.2	Q4
19. Journal of Himalayan Earth Sciences	0.2	Q4	0.4	Q4

***Note:*** Data recorded from the Clarivate Report 2025;^1^ calculation based on the average citation indicators and JIF percentile; journals with suppressed information were not included.

In the medical and health sciences, the Pakistan Journal of Medical Sciences has achieved an impact factor (IF) of 1.7, with a quartile ranking of Q2. This indicates that the journal is publishing high-quality research, garnering more citations with an increasing trend in its impact factor. The cornerstone of any nation’s progress and prosperity is based on education and research, which establish the foundation for a progressive future. Nations that invest in academia and research and foster a culture of innovation tend to experience accelerated growth and an improved quality of life. However, countries that neglect these areas often find themselves mired in poverty, social strife, and stagnation. The path to Pakistan’s national interest is paved with the bricks of education, research, and development, grounded in a merit-based culture within universities and research institutes. These elements are essential for the progress and prosperity of nations.^3^

**Fig.1:**
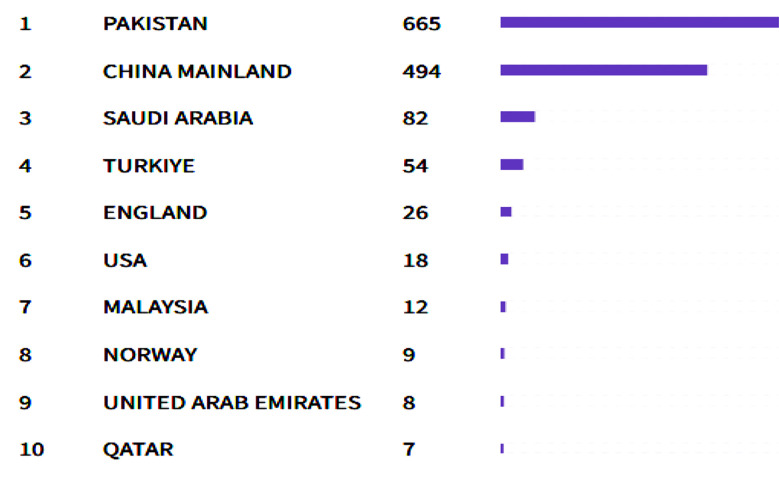
Contributions by country/region that have contributed the most papers to the journal in the recent three years.

**Fig.2:**
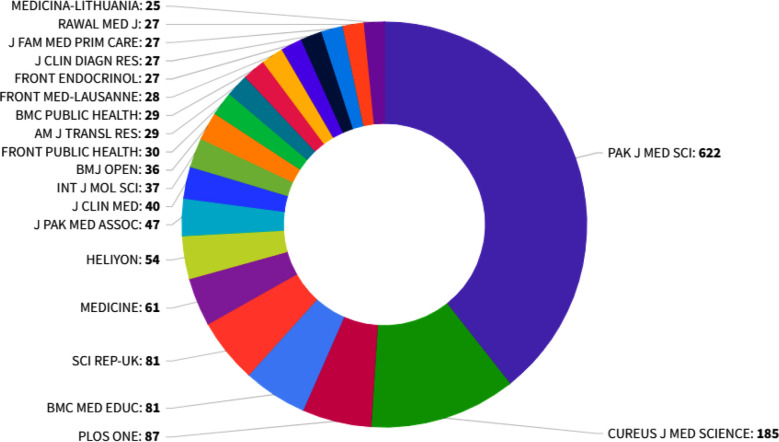
Top 20 Journals citing the Pk J Med Sci. A Critical Look at the Latest Impact Factor Rankings

The Pakistan Journal of Medical Sciences received articles from across the globe, including Pakistan, China, Türkiye, the United Kingdom, the USA, Malaysia, and the United Arab Emirates (Fig.1). Worldwide, the leading Science journals, including BMJ, Frontiers, BMC, PLOS ONE, cited the Pak J Med Sci. Hence, the journal’s Impact Factor and Quartile ranking increased. It indicates the rising standing of Pak J Med Sci in the global scientific community (Table-I, Fig.2).

A critical examination of the latest Impact Factor (IF) data reveals that while the Pakistan Journal of Medical Sciences has shown a significant improvement in its IF—from 1.2 to 1.7—the Journal of the Pakistan Medical Association (JPMA) has maintained its IF at 0.8. The Journal of the College of Physicians and Surgeons Pakistan (JCPSP) has seen a slight improvement, increasing its IF from 0.7 to 0.8. However, no other medical journal from Pakistan has been able to secure inclusion in the Science Citation Index Expanded (SCIE), part of the Web of Science Core Collection.

Nevertheless, 19 other journals from Pakistan, including eight medical journals, succeeded last year in being included in the Emerging Source Citation Index (ESCI), which is considered a promising start. Among these, Rawal Medical Journal and Gomal Medical Journal failed to maintain their previous IFs, with Rawal’s IF dropping from 0.4 to 0.2, and Gomal’s from 0.5 to 0.1. Others—such as the Annals of King Edward Medical University (KEMU), Pakistan Heart Journal, Annals of Abbasi Shaheed Hospital/Karachi Medical & Dental College, Journal of Liaquat University of Medical & Health Sciences, Khyber Medical University Journal—have maintained their previous IFs, which range from 0.1 to 0.2 depending on the journal. Anesthesia Pain and Intensive Care has improved its IF from 0.2 to 0.3. (Table-I).

It is worth mentioning that journalology has now emerged as an important discipline with various sub-specialties. Publishing a high-quality, peer-reviewed journal is a demanding and often frustrating task that requires teamwork and consistent institutional support.^4^

The reasons behind the current state of affairs are not difficult to identify. Most journals published by medical institutions are managed by editors working on an honorary basis. Typically, a faculty member is given the additional responsibility of serving as the editor, but there is often no proper infrastructure, dedicated support staff, or functioning editorial system in place. The retirement or transfer of such a faculty member can severely disrupt the functioning of the journal. Moreover, the interest and involvement of the institution’s leadership also play a crucial role in the journal’s success.

The solution is straightforward. Medical institutions must establish a formal editorial structure with a properly equipped office, trained support staff, and a sustainable workflow (some institutions have recently achieved this). Being a good physician or surgeon does not necessarily qualify one to be an effective editor. Medical journalism is both a science and art. Healthcare professionals are well-versed in science, but they must learn the art of editing a scientific journal.^5^

Efforts must also be made to train editorial staff so that the retirement or transfer of an individual does not disrupt journal operations. Editors should receive financial incentives and be appointed on a contractual basis with clearly defined roles, responsibilities, and term limits. This would allow them to understand the duration and expectations of their editorial role. If their performance is satisfactory, their contracts could be renewed. Currently, editors face uncertainty, not knowing if they will be replaced without notice, as the administration might arbitrarily assign the role to another faculty member.

**Improving the Standard of Medical Journalism in Pakistan: A Collaborative Effort**

PAME (Pakistan Association of Medical Editors), during its 2016 conference organized at the University of Health Sciences (UHS), held a special session on “Professionalism and Medical Journal Editors.” Since its inception in 2010, PAME has been actively organizing hands-on workshops on scientific writing, peer review, and training sessions for biomedical journal editors.

In 2019, in collaboration with UHS, PAME launched a six-month Certificate Course in Medical Editing, which was later followed by another six-month Certificate Course in Medical Journalism and Editing. So far, over 200 healthcare professionals have successfully completed these courses. Currently, the 7th batch of the Medical Editing certificate course and the 3rd batch of the Medical Journalism and Editing course are undergoing training.^6^

The next planned step is the launch of a Diploma in Medical Journalism, with the ultimate goal of establishing a Master’s program in Medical Journalism. The UHS administration, especially Vice Chancellor Prof. Ahsan Waheed Rathore and Pro Vice Chancellor Prof. Nadia Naseem, has shown keen interest in these initiatives. A state-of-the-art Department of Medical Journalism is scheduled to be inaugurated at UHS in July 2025.^7^

In addition, PAME, in collaboration with Core Teach/KEMU, has launched an Online Course in Medical Editing and Journalism (CMEJ), now in its sixth cohort. This initiative was introduced to accommodate individuals who were unable to attend the in-person course at UHS for various reasons. Many healthcare professionals who have completed these courses have reported significant benefits.^8^

The collaborative efforts of UHS and PAME—supported by over 25 distinguished researchers and medical editors from Pakistan, Iran, and Saudi Arabia who serve as facilitators and mentors—have significantly contributed to enhancing the standards of medical journalism in the country, which is truly commendable.^7^

The Pakistan Medical & Dental Council (PM&DC), through its Journal Evaluation Committee, comprising many senior medical editors, has also played a vital role in evaluating and recognizing biomedical journals. Each journal is assessed by two to three editors, with detailed deficiencies noted and communicated to the editors for correction before re-evaluation.^9^ Despite facing considerable pressure, PM&DC rightly refused to recognize more than half a dozen journals, some of which had long been in publication and were even recognized by other regulatory bodies. However, most of these journals lacked understanding of the peer review process and did not implement any formal peer review systems.^10^

The President of PM&DC has been supportive and receptive to the views of committee members, and successive chairpersons of the Journals Committee have also contributed effectively. The Higher Education Commission (HEC) is another regulatory body responsible for journal recognition. However, since HEC oversees numerous disciplines, medicine is just one among many, and unfortunately, it lacks the necessary expertise in this field. As a result, its performance in evaluating medical journals has been suboptimal. Except for one or two members, the committee lacks domain-specific knowledge, which has led to the recognition of several predatory journals, causing embarrassment when these journals are later delisted from international databases. There is a need for collaboration between PM&DC and HEC for evaluation and recognition of biomedical journals.

The shared objective of all stakeholders—HEC, PM&DC, and PAME—is to improve the quality of published manuscripts, enhance the standards of medical journals, and increase Pakistan’s contribution to global medical literature, thereby promoting a robust research culture. While intentions are positive across the board, there is a critical need for coordination, collaboration, and closing communication gaps among these organizations. This suggestion has been made repeatedly in the past.^5^ Let us renew our collective efforts to turn this vision into reality—for the national interest and the advancement of medical research in Pakistan.

Research in science and the social sciences plays a pivotal role in education, planning, decision-making, and economic progress, alongside long-standing and sustainable development. It improves living standards and the quality of life. Academic journals are the primary source for the dissemination of knowledge, discoveries, and innovations. The scientific publishing culture is essential for the advancement and sustainable development of nations.

Pakistan must enhance the academic, scientific and research culture and promote academic journals, and for research, sustainable development through knowledge dissemination and innovation.
